# RNF216 Regulates the Migration of Immortalized GnRH Neurons by Suppressing Beclin1-Mediated Autophagy

**DOI:** 10.3389/fendo.2019.00012

**Published:** 2019-01-24

**Authors:** Fangfang Li, Dengfeng Li, Huadie Liu, Bei-Bei Cao, Fang Jiang, Dan-Na Chen, Jia-Da Li

**Affiliations:** ^1^Center for Medical Genetics, School of Life Sciences, Central South University, Changsha, China; ^2^Hunan Key Laboratory of Animal Models for Human Diseases, Central South University, Changsha, China; ^3^Department of Basic Medical Sciences, Changsha Medical University, Changsha, China

**Keywords:** hypogonadotropic hypogonadism, GnRH neuron, RNF216, migration, autophagy

## Abstract

*RNF216*, encoding an E3 ubiquitin ligase, has been identified as a causative gene for Gordon Holmes syndrome, characterized by ataxia, dementia, and hypogonadotropic hypogonadism. However, it is still elusive how deficiency in *RNF216* leads to hypogonadotropic hypogonadism. In this study, by using GN11 immature GnRH neuronal cell line, we demonstrated an important role of RNF216 in the GnRH neuron migration. RNA interference of RNF216 inhibited GN11 cell migration, but had no effect on the proliferation of GN11 cells or GnRH expression. Knockdown of RNF216 increased the protein levels of its targets, Arc and Beclin1. RNAi of Beclin1, but not Arc, normalized the suppressive effect caused by RNF216 knockdown. As Beclin1 plays a critical role in the autophagy regulation, we further demonstrated that RNAi of RNF216 led to increase in autophagy, and autophagy inhibitor CQ and 3-MA rescued the GN11 cell migration deficit caused by RNF216 knockdown. We further demonstrated that pharmacological increase autophagy by rapamycin could suppress the GN11 cell migration. We thus have identified that RNF216 regulates the migration of GnRH neuron by suppressing Beclin1 mediated autophagy, and indicated a potential contribution of autophagy to the hypogonadotropic hypogonadism.

## Introduction

The GnRH neurons in the hypothalamus secrete gonadotropin releasing hormone, which control the production and release of the gonadotropin-luteinizing hormone (LH) and follicle-stimulating hormone (FSH). LH and FSH stimulate gametogenesis and sex steroid production in the gonads (1). During embryonic development, GnRH neurons migrate from the olfactory placode, through the nasal mesenchyme, to reach their final destination in the basal forebrain (2, 3). Disruption of the genesis, migration of GnRH neurons and/or synthesis, secretion and signaling of GnRH in humans can lead to congenital hypogonadotropic hypogonadism (CHH) (1, 4, 5). The incidence of CHH is about 1/4,000 in men and about 1/20,000 in women (6).

CHH is a disorder with great heterogeneity in genetics and phenotype (6). About 40 different genes have been associated with CHH, only accounting for ~50% patients (7, 8). RNF216 (ring finger protein 216), an E3 ubiquitin ligase, can mediates ubiquitination and degradation of its target through ubiquitin-proteasome system. Margolin et al. recently identified *RNF216* mutations in Gordon Holmes syndrome, characterized by ataxia, dementia, and hypogonadotropic hypogonadism (9). And deficiency in *RNF216* led to smaller testis and abnormal testis development in mice (10). However, the pathological mechanism is still unknown.

In this study, by using GN11 immature GnRH neuronal cell line, we demonstrated that RNF216 regulates the GnRH neuron migration by suppressing Beclin1-mediated autophagy.

## Results

### RNA Interference (RNAi) of RNF216 Inhibited GN11 Cells Migration

To study the effect of RNF216 on the proliferation and migration of GnRH neurons, we utilized the GN11 immature GnRH neuron cell line (11), which is derived by limited dilution and cloning of an olfactory tumor from a mouse bearing a human GnRH-simian virus 40 T antigen transgene (12).

**Figure 1:**
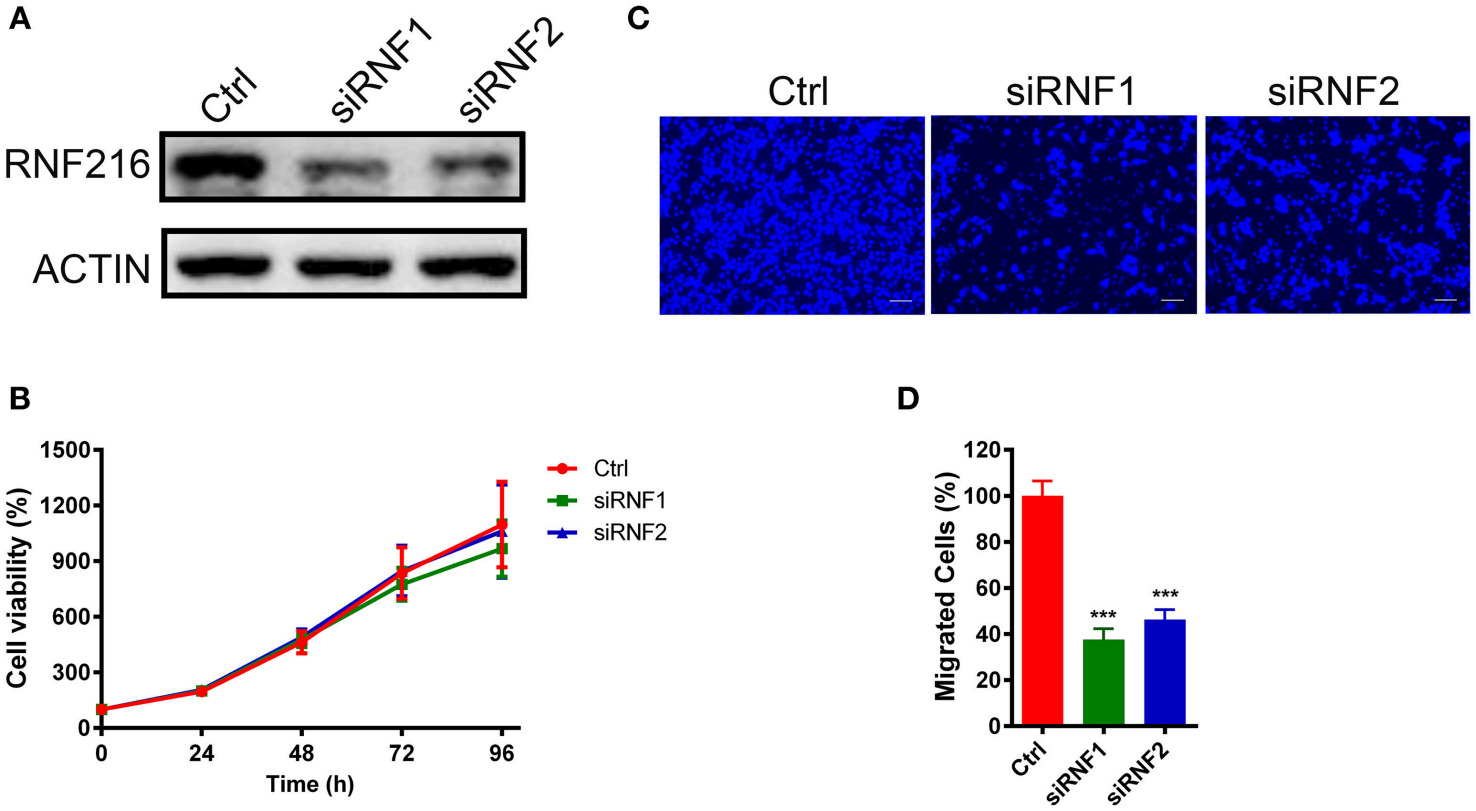
Depletion of RNF216 inhibited GN11 cells migration. **(A)** Depletion of endogenous RNF216 with small interference RNAs (siRNAs). GN11 cells were transfected with control siRNA (siNC) or RNF216-specific siRNAs (siRNF1-2). RNAi efficiency was confirmed by immunoblotting of the endogenous protein levels. ACTIN was used as a loading control. **(B)** Depletion of RNF216 has no effect on cell proliferation. GN11 cells were transfected with siNC or siRNF, cell proliferation, and viability were examined with an MTT assay at 0, 24, 48, 72, and 96 h after transfection. The quantitative data were calculated from three independent experiments, and were shown as mean ± SEM. **(C)** Representative images of GN11 cells from transwell assays. Scale bar = 50 μm. **(D)** Depletion of RNF216 decreased the number of migrated GN11 cells. Data is shown as the mean ± SEM of three independent experiments, ^***^P < 0.001, unpaired *t-*test.

We first down-regulated the RNF216 expression in GN11 cells using small interfering RNAs (siRNAs). As shown in Figure 1A, both siRNAs efficiently downregulated the expression of *RNF216*. We then used a 3-(4, 5-dimethyl-2-thiazolyl)-2, 5-diphenyl-2-H-tetrazolium bromide (MTT) assay to see the effect of RNF216 knockdown on the GN11 cell proliferation. As shown Figure 1B, RNAi of RNF216 did not influence the GN11 cell proliferation. Knockdown of RNF216 had no effect on the expression of GnRH, either (Figure S1).

We further used a trans-well assay to study the effect of RNF216 knockdown on the GN11 cells migration. Cells transfected with a control siRNA or siRNAs targeting RNF216 were loaded onto the upper chamber, and cells migrated to the bottom layer of the chamber were stained with DAPI and counted. As shown in Figures 1C,D, RNAi of RNF216 significantly reduced the cells migrated to the bottom layer of the chamber.

### RNF216 Regulated GN11 Cells Migration Independent of Arc

As an E3 ubiquitin-protein ligase, RNF216 mediates ubiquitination of multiple molecules, such as TLR4, RIP1, TRAF3, Arc, and Beclin1 (13–17). Husain et al. recently demonstrated that Gordon Holmes syndrome-associated RNF216 mutations lead to synaptic and cognitive impairments via Arc mis-regulation (18). As Arc is a cytoskeletal protein associated with cell migration (19), we first studied the effect of Arc on the regulation of RNF216 of GN11 cell migration. Indeed, RNAi of RNF216 upregulated significantly the protein level of Arc (Figure S2A). We then sought to see if downregulation of Arc (Figure S2B) could rescue the GN11 cell migration caused by RNF216 knockdown. As shown in Figures S2C,D, the GN11 cell migration was equally suppressed by RNAi of RNF216 regardless of Arc levels. Therefore, RNF216 regulated GN11 cells migration independent of Arc.

### RNF216 Regulated GN11 Cells Migration via Beclin1

RNF216 was also able to mediate the ubiquitination and degradation of autophagy-related protein Beclin1, inhibiting the occurrence of autophagy (17). Wang et al. demonstrated that RNF216 can promote colorectal cancer cell proliferation and migration by inhibiting Beclin1-dependent autophagy (20). We then sought to determine whether RNF216 regulates the migration of GN11 cells through Beclin1.

**Figure 2:**
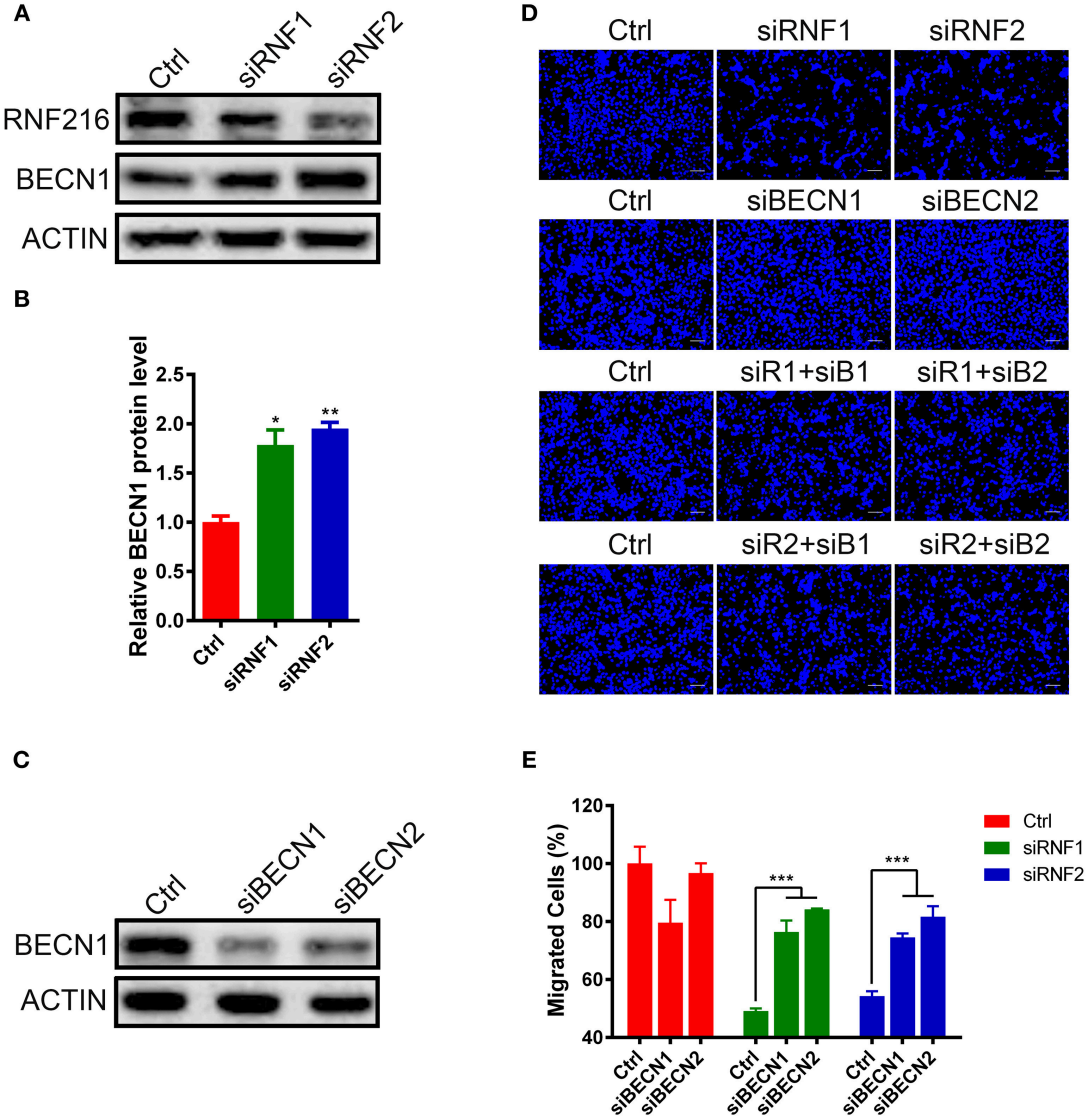
RNF216 regulated GN11 cells migration via Beclin1. **(A)** Depletion of RNF216 upregulated Beclin1 protein level in GN11 cells. Beclin1 protein was detected in GN11 cells transfected with siNC or siRNF by immunoblotting. ACTIN was used as a loading control. **(B)** Quantification of Beclin1 protein levels in GN11 cells as detected by immunoblotting. Data is shown as the mean ± SEM of three independent experiments, *P < 0.05, **P < 0.01. **(C)** Efficient depletion of endogenous Beclin1 with siRNAs. **(D)** Representative images of GN11 cells from transwell assays with various treatment. Scale bar = 50 μm. **(E)** Depletion of Beclin1 rescued the impaired GN11 cells migration induced by RNAi of RNF216. Data is shown as the mean ± SEM of three independent experiments, ***P < 0.001, two way ANOVA.

We first measured the Beclin1 protein level after knockdown of RNF216 in GN11 cells. As shown in Figures 2A,B, RNAi of RNF216 significantly increased the Beclin1 protein level. We then investigated the GN11 cell migration after knockdown of both Beclin1 and RNF216. While knockdown of RNF216 alone decreased the GN11 cell migration, knockdown of Beclin1 (Figure 2C) significantly rescued the migration deficit caused by RNAi of RNF216. Knockdown of Beclin1 alone has no effect on the GN11 cell migration (Figures 2D,E). These data indicated that RNF216 regulated GN11 cells migration via downregulation of Beclin1.

### RNF216 Regulated GN11 Cells Migration Through Autophagy

Beclin1 plays an essential role in autophagy induction (21–23), we then assessed autophagy in RNF216-depleted GN11 cells by measuring autophagy marker light chain 3 (LC3) and P62 protein under starvation stimulation.

**Figure 3:**
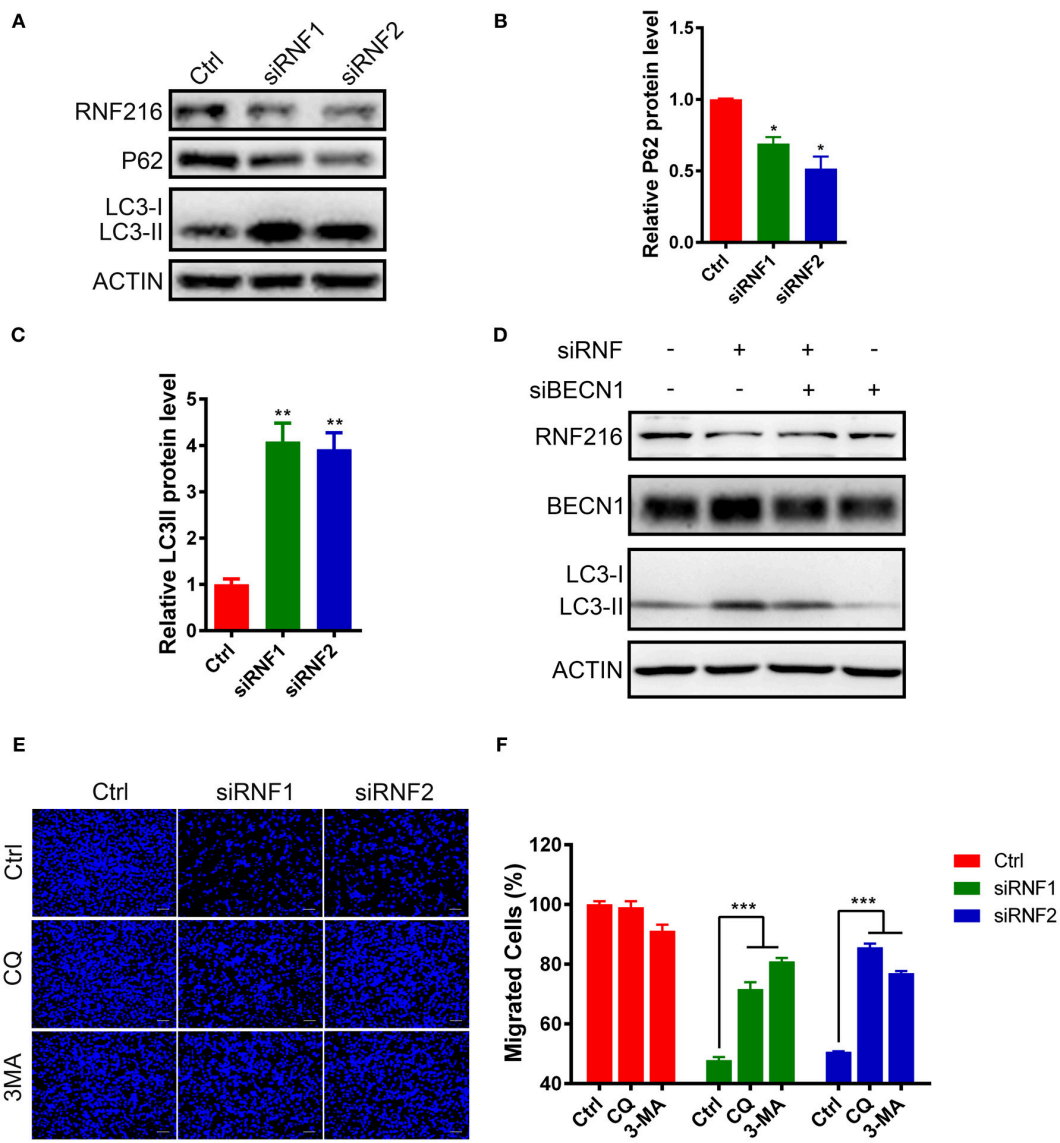
RNF216 regulated GN11 cells migration through autophagy. **(A)** Depletion of RNF216 upregulated autophagy flux in GN11 cells. The protein levels of LC3 and P62 were detected with immunoblotting in GN11 cells transfected with siNC and siRNF. ACTIN was used as a loading control. **(B,C)** Quantification of LC3-II **(B)** and P62 **(C)** protein levels in GN11 cells as detected by immunoblotting. Data is shown as the mean ± SEM of three independent experiments, *P < 0.05, **P < I0.01, unpaired *t-*test. **(D)** Knockdown of Beclin1 normalized the LC3-II protein level induced by RNF216 deficiency. The protein levels of LC3 were detected with immunoblotting in GN11 cells transfected with respective siRNAs. ACTIN was used as a loading control. **(E)** Representative images of GN11 cells from transwell assays with various treatment. Scale bar = 50 μm. 3-MA (24 μM) and CQ (40 nM) were used. **(F)** Inhibition of autophagy rescued the impaired GN11 cells migration induced by RNAi of RNF216. Data is shown as the mean ± SEM of three independent experiments, ***P < 0.001, two way ANOVA.

The LC3 antibody used in this study can only detect LC3-II in the GN11 cells, but can detect both LC3-I and LC3-II in 293T cell (Figure S3). As shown in Figures 3A–C, RNF216-depletion significantly induced LC3-II in the GN11 cells. Furthermore, RNF216-depletion also led to significant decrease in P62 protein level.

To see the involvement of Beclin1 in the autophagy induced by RNF216-depletion, we measured the protein levels of LC3 in GN11 cells transfected with siRNAs targeting RNF216 and Beclin1. As shown in Figure 3D, knockdown of Beclin1 normalized the LC3-II protein level induced by RNF216 deficiency, whereas RNAi of Beclin1 led to downregulation of LC3-II protein level.

Autophagy plays an important role in regulating the physiological function of cells, including cell migration (24). To see if increased autophagy influx in the RNF216-depleted GN11 cells is responsible for the deficient migration, the migration of RNF216-depleted GN11 cells was monitored with autophagy inhibitors 3-MA and CQ. As shown in Figures 3E,F, both 3-MA and CQ significantly reversed the migration deficiency in RNF216-depleted GN11 cells. Our results thus suggested that RNF216 regulated GN11 cells migration by inhibiting autophagy flux.

### Upregulation of Autophagy Inhibited GN11 Cells Migration

**Figure 4:**
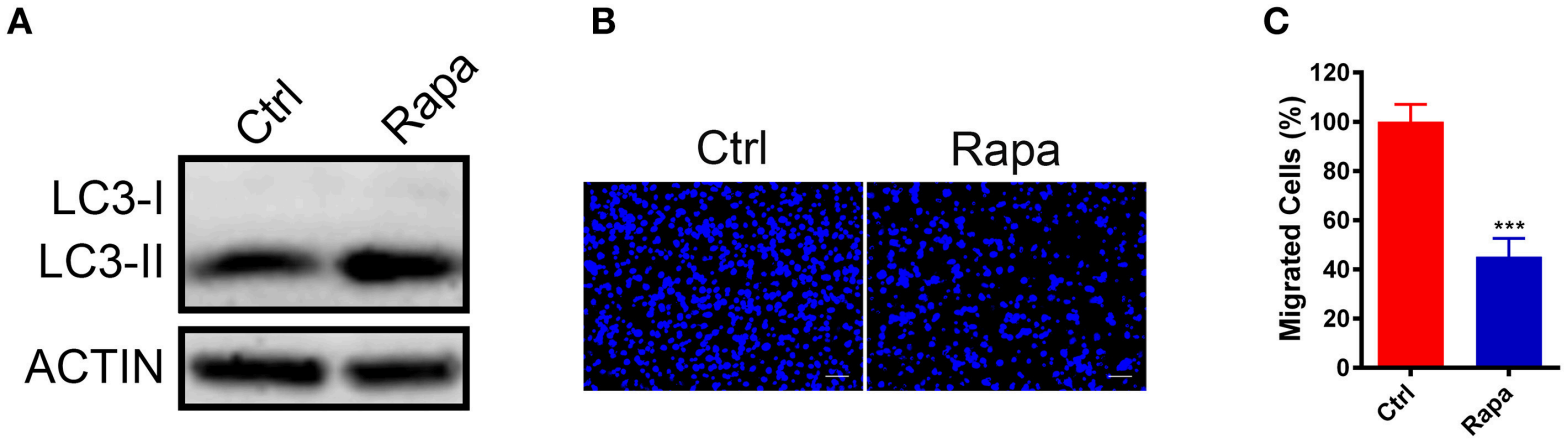
Upregulation of autophagy inhibited GN11 cells migration. **(A)** The promotion of autophagy was confirmed by immunoblotting. ACTIN was used as a loading control. **(B)** Representative images of GN11 cells from transwell assays with Rapamycin (500 nM) treatment. Scale bar = 50 μm. **(C)** Rapamycin (500 nM) inhibited GN11 cells migration. Data is shown as the mean ± SEM of three independent experiments, ***P < 0.001, unpaired *t*-test.

To further investigate if increased autophagy flux is sufficient to halt the GN11 cells migration, we treated GN11 cells with an autophagy activator rapamycin for 30 h and the cell migration was monitored with a trans-well assay. The promotion of autophagy was confirmed by immunoblotting (Figure 4A). As shown in Figures 4B,C, the migration was decreased significantly in the rapamycin-treated GN11 cells compared with vehicle-treated cells.

## Discussion

RNF216, an E3 ubiquitin ligase, can regulate the ubiquitination level of many substrates to participate in various physiological activities. In this study, we demonstrated that depletion of RNF216 disrupted the migration, but had no effect on the proliferation of GnRH neuronal cell line. This effect seems to be mediated by the Beclin1-regulated autophagy. We further showed that increasing autophagy *per se* suppressed the migration of GnRH neuronal cell line, suggesting the involvement of autophagy in the pathogenesis of hypogonadotropic hypogonadism.

The CHH-associated genes are involved in the genesis, migration of GnRH neurons and/or synthesis, secretion and signaling of GnRH (4). For instance, Anosmin-1, encoded by CHH-causative gene KAL1, regulates the migration of GnRH neuron from the olfactory placode to the hypothalamus. KISS1/KISS1R signaling triggers the GnRH release, whereas the gonadotrope stimulation is mediated by GNRH1/GNRHR signaling (25). Nevertheless, many genes may be involved in more than one processes. For example, FGF8/FGFR1 signaling not only participates in the early emergence of GnRH neurons from the embryonic olfactory placode (26), but also regulates the initial specification and long-term survival of GnRH neurons (27–29). Furthermore, decreased FGF8/FGFR1 signaling disrupts the olfactory bulb morphogenesis, leading to defects in GnRH neuronal migration (30). In this study, by using an immortalized GnRH cell line, we demonstrated that RNF216 regulated the GnRH neuron migration, but has no effect on the cell proliferation and GnRH expression. As RNF216 is also expressed in the mature GnRH cell line GT1-7 (data not shown), it will be intriguing to investigate if RNF216 regulates the homeostasis of GnRH neuron and/or GnRH secretion.

Autophagy is a biological process that maintains cell homeostasis and physiological functions of cells (31, 32). When cells are disturbed by external environment, they can form independent double membrane structure, which extend continuously to form autophagosome. Autophagosome, containing segmental cytoplasm, degraded organelles and protein, fuse with lysosomes to form autolysosome, in which the inclusion degrade so as to adapt with outside stress (33). Autophagy has been demonstrated to regulate cell migration in a cell type-dependent manner; it inhibits the migration of some cells, but stimulates the migration of other cells. Autophagy may affect the migration of malignant glioma cells by impacting the epithelial-mesenchymal transformation regulator SNAIL and SLUG (34); whereas autophagy influences the macrophage migration by regulating the degradation of guanine nucleotide exchange factor (35). Moreover, autophagy may also regulate cell migration by influencing the assembly of cytoskeletal proteins, the formation of focal adhension, the production of cytokines, and energy metabolism.

Autophagy initiation is coordinated by two kinases, unc-51 like kinase 1 (ULK1, also known as autophagy-related (ATG)-1) and vacuolar protein sorting-34 (VPS34, also known as PIK3C3). Activation of both ULK1 and VPS34 drives the recruitment of additional ATG proteins to phagophore membranes and promotes autophagosomal maturation. VPS34 complex is mainly composed of VPS34, Beclin1, and ATG14. Beclin1 governs the autophagic process by regulating PI3KC3-dependent generation of PI3P and the subsequent recruitment of additional ATG proteins that orchestrate autophagosome formation (36, 37). On the other hand, ULK1 functions in a complex with ATG13, and focal adhesion kinase family interacting protein of 200 kDa (FIP200). mTOR plays a critical role in the autophagy initiation through ULK1, and inhibition of mTOR by rapamycin enhances the kinase activity of ULK1. mTOR may inhibit ULK1 through direct protein phosphorylation. Phosphorylation of Atg13 by mTOR reduced the affinity of Atg13 for Atg1 to prevent autophagy, as the Atg1-Atg13 association and subsequent activation of Atg1 are required for autophagy induction. In addition, mTOR may indirectly destabilize ULK1 and impairs autophagy through phosphorylation of Autophagy/Beclin-1 regulator 1 (AMBRA1). Phosphorylation of AMBRA1 by mTOR prevents Lys-63-linked ubiquitination of ULK1, which causes self-association and enhances stability of ULK1 (23, 36, 38).

In this study, we found that autophagy, elicited by Beclin1 (RNF216 depletion) or mTOR inhibition (rapamycin treatment), suppressed the migration of GnRH neuronal cell line. It will be intriguing to understand the molecular mechanisms underlying the inhibitory effect of autophagy on GnRH cell migration. Our results also imply that increased autophagy by genetic mutations and/or environmental factors may contribute to the pathogenesis of hypogonadotropic hypogonadism. Genetic studies in large CHH cohorts, combined with cell/animal model studies, may identify more autophagy-related genes to be associated with CHH.

## Materials and Methods

### Cell Culture

GN11 cell lines was gifted from Professor Sally Radovick and Horacio Novaira. Cells were cultured in Dulbecco's modified Eagle's medium (DMEM; Hyclone, Logan, UT, USA) containing 15% fetal bovine serum (Gibco, Grand Island, NY, USA), and 25 mM glucose, 5 mM l-glutamine,100 mg/ml streptomycin, 100 U/ml penicillin (Gibco, Grand Island, NY, USA) in humidified air at 37°C with 5% CO2. GN11 cells were plated between fourth and tenth passages. GN11 cells were passaged when reach to 90% confluence.

### Cell Transfection

GN11 cells were plated at 50% confluence and transfected with siRNAs (50 nM) specific to RNF216, BECN1, and Arc (GenePharma, Halley Road, Shanghai, P. R. China) using Lipofectamine 2000 (Invitrogen, Carlsbad, CA, USA) according to the manufacturer's instructions. Control cells were transfected with a non-targeting siRNA (50 nM) (GenePharma, Halley Road, Shanghai, P. R. China). All treatments were performed in Reduced-Serum Medium (Opti-MEM; Gibco, Grand Island, NY, USA). The transfected cells were cultured in reduced-serum medium for 6 h, then the medium was replaced by complete medium. After 48 h, cells were harvested for immunoblotting or subsequent assays. The effective siRNA sequences are as following:

siRNF216-1: sense: 5′-GCAGACAGCAGACGAUAUUTT−3′, antisense: 5′- AAUAUCGUCUGCUGUCUGCTT-3′), siRNF216-2: sense (5′- GCUUGAAGACCAGCAGUUATT-3′), antisense(5′- UAACUGCUGGUCUUCAAGCTT-3′).

siBECN1-1: sense (5′-GGUACCGACUUGUUCCCUATT-3′), antisense(5′- UAGGGAACAAGUCGGUACCTT-3′), siBECN1-2: sense (5′- GCUCCAUGCUUUGGCCAAUTT-3′), antisense(5′- AUUGGCCAAAGCAUGGAGCTT-3′).

siArc-1: sense (5′-GCUCAGCAAUAUCAGUCUUTT-3′), antisense(5′- AAGACUGAUAUUGCUGAGCTT-3′), siArc-2: sense (5′- CCAGGAAGCUGAUGGCUAUTT-3′), antisense(5′- AUAGCCAUCAGCUUCCUGGTT-3′).

### Cell Proliferation Assay

The proliferation assay used a colorimetric assay based on measuring the reduction of 3-(4,5-dimethyl-2-thiazolyl)-2,5-diphenyl-2-H-tetrazolium bromide (MTT; Sigma, St. Louis, MO, USA). GN11 cells were plated in a 96-well plate with 1,000 cells each well and cultured in complete medium. MTT assay was used to measure viable proliferating cells at 0, 24, 48, 72 and 96 h after plating. At the end of each experiment, 20 μL of the 5 mg/mL MTT solution was added to each well and incubated for 4 h at 37°C. After incubation, the medium were replaced with 100 μl DMSO (Dimethyl sulfoxide; Sigma, St. Louis, MO, USA) each well and slowly shaked for 10 min at room temperature. Absorbance was measured at 570 nm using a spectrophotometer. The experiments for each assay were performed with 8 replicates for each treatment condition and repeated 3 times using different cell passages.

### Western Blot Analysis

When GN11 cells reach to 90% confluence, total protein lysates were harvested from cells with lysis buffer (150 mM NaCl, 50 mM PH7.5 Tris-HCl, 10% glycerol, 4% sodium dodecyl sulfate and protease inhibitor cocktail [Sigma, St. Louis, MO, USA]). Cell lysates were boiled at 100°C for 10 min and then supernatant (10 μg) was subjected to sodium dodecyl sulfate–polyacrylamide gel electrophoresis (SDS-PAGE) after centrifugation (13,000 g for 10 min). After electrophoresis, the proteins were transferred onto polyvinylidene difluoride membranes. The membranes were blocked with 5% fat-free milk in TBS with 0.1% Tween-20 for 1 h. The primary antibodies used were as following: 1:2,000 rabbit anti-RNF216 (Sigma, St. Louis, MO, USA); 1:2,000 rabbit anti-BECN1 (Cell Signaling Technology, Danvers, MA, USA); 1:4,000 rabbit anti-P62 (Cell Signaling Technology, Danvers, MA, USA); 1:1,000 rabbit anti-LC3 (Cell Signaling Technology, Danvers, MA, USA). Primary antibodies were incubated at 4°C overnight. After washing, HRP-conjugated secondary antibodies were incubated at room temperature for 1 h. After washing, the blots were visualized with a chemiluminescent signal (Immobilon Western HRP Substrate Peroxide Solution; Sigma, St. Louis, MO, USA) and subsequently digitized (Fusion Solo; Vilber Lourmat, Paris, France) and analyzed (Image J; National Institutes of Health, Bethesda, Maryland, USA). The detection of every protein was performed at least 3 times.

### Transwell Assay

Transwell assay was used to characterize GN11 cellular migration. After GN11 cells with siRNA treatment for 36h, the cells were harvested with 0.25% Trypsin-EDTA (Gibco, Grand Island, NY, USA), centrifuged for 5 min at 800 g, and resuspended in serum-free medium. GN11 cells (5 × 10^4^ cells) were seeded onto the upper compartment of the well (Costar, Kennebunk, ME, USA) separated by 8 mm-pore filters with 200 μL serum-free DMEM. The lower compartment contained 500 μL complete medium. After incubation at 37°C with 5% CO2 for 24 h, cells on the upper side of the filters were then mechanically removed. GN11 cells at the lower side of the filter were then fixed in cold 100% methanol for 30 min and washed twice using PBS. Subsequently, the nuclei were stained with DAPI (Sigma, St. Louis, MO, USA) for 30 min and then washed twice using PBS. Images were acquired under a microscope (LEICA, Germany) and stained cells were counted in five fields to determine the average number of cells that had migrated. The experiments for each assay were performed with three replicates and repeated 3 times.

### Quantitative RT-PCR (qPCR)

The efficiency of RNF216 silencing by siRNA and GnRH expression were measured in GN11 cells by qPCR. Total RNA was harvested 48 h after transfection with siRNA. Then 1 μg of RNA was reverse transcribed using the RevertAid First Strand cDNA Synthesis Kit, and the cDNA was analyzed by qPCR using Maxima SYBR Green qPCR Master Mix (Thermo Fisher Scientific, Waltham, Massachusetts, U.S.) with a 10 μM concentration of the appropriate primer pair. The primers used to amplify RNF216 were sense primer, 5′-GCCCATCCTCTAGGAGAGCTT-3′, and antisense primer, 5′-CCGTTTCTTTCACTAACAGTGGA-3′. The primers used to amplify GnRH were sense primer, 5′-AGCACTGGTCCTATGGGTTG-3′, and antisense primer, 5′-GGGGTTCTGCCATTTGATCCA-3′. The primers used to amplify Gapdh were sense primer, 5′-TGGATTTGGACGCATTGGTC-3′, and antisense primer, 5′-TTTGCACTGGTACGTGTTGAT-3′. All samples were assayed in three duplicate using the LightCycler® 96 System (Roche, Basel, Switzerland). The qPCR conditions were as follows: initial denaturation and enzyme activation at 95°C for 10 min followed by 40 cycles of denaturation (95°C, 15 s), annealing, and reading (60°C, 30 s). Melt curve analyses were conducted after each qPCR to demonstrate the presence of a single amplicon. Relative expression of genes was calculated by the 2^−ΔΔCt^ method and normalized to the housekeeping gene Gapdh. This experiment was repeated 3 times.

### Statistical Analysis

The data was analyzed using Prism6 software (GraphPad Software, San Diego, CA, USA). Statistical significance were evaluated by unpaired *t*-test in two independent groups. Multiple treatment groups were analyzed using ANOVA compared within individual experiments. Data are shown as mean ± SEM. A *P* < 0.05 was considered to indicate a significant difference.

## Author Contributions

FL and DL contributed equally to this work. J-DL conceived and designed the study, performed data analysis and data interpretation, and wrote the manuscript. D-NC designed the study and performed data analysis. FL and DL performed all the experiments. HL provided the cells and techniques. B-BC provided technical assistance and helped to process the manuscript. FJ provided instrument for performing experiment and participated in writing the manuscript.

### Conflict of Interest Statement

The authors declare that the research was conducted in the absence of any commercial or financial relationships that could be construed as a potential conflict of interest.
