# Supplementary material for: RNF216 Regulates the Migration of Immortalized GnRH Neurons by Suppressing Beclin1-Mediated Autophagy
Source: Front Endocrinol (Lausanne). 2019 Jan 24;10:12. doi: 10.3389/fendo.2019.00012 (PMC6354547; doi:10.3389/fendo.2019.00012)
Supplement: Supplementary file 1 [file Data_Sheet_1.docx]

***Supplementary Material***

**RNF216 regulates the migration of immortalized GnRH neurons** **by suppressing Beclin1-mediated autophagy**

**Fangfang Li^1, 2†^, Dengfeng Li^1, 2†^, Huadie Liu^1, 2^, Bei-Bei Cao^1, 2^, Fang Jiang^1, 2^, Dan-Na Chen^3^*, Jia-Da Li^1, 2^***

**^†^These authors contributed equally to this work.**

*** Correspondence author:** E-mail address: [chendanna007@gmail.com](mailto:chendanna007@gmail.com) (D-N Chen). E-mail address: lijiada@sklmg.edu.cn (J-D Li).

**Supplementary Figures**

**
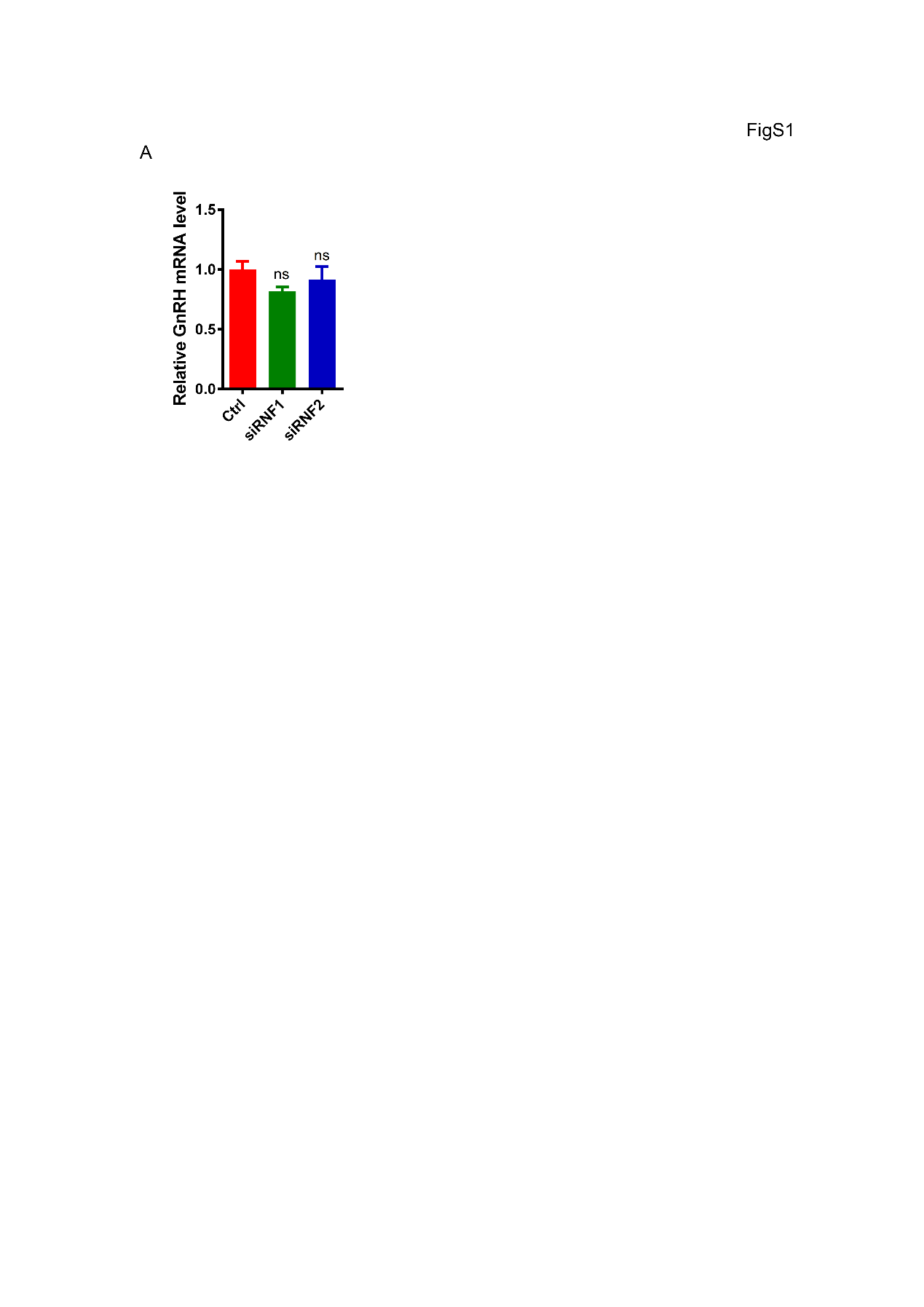
**

**Figure S1: RNF216 has no effect on GnRH expression in the GN11 cells.** GN11 cells were transfected with control siRNA or siRNAs targeting RNF216 (siRNF1-2). The mRNA level of GnRH was detected by qPCR. Relative expression of GnRH was calculated by the 2^-△△Ct^ method and normalized to the housekeeping gene Gapdh. Data is shown as the mean ± SEM of three independent experiments, ns present no significantly, unpaired *t test*.

**
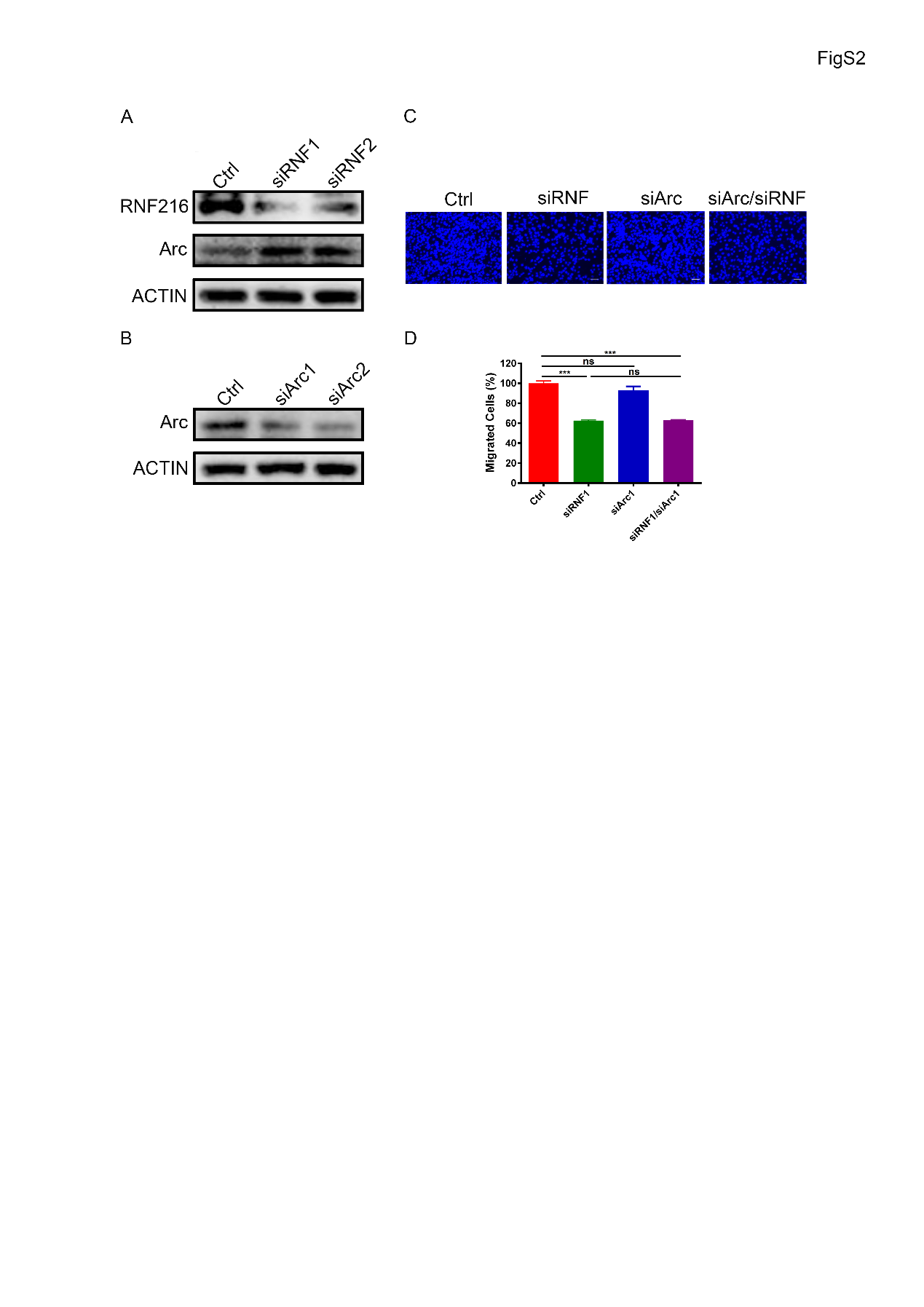
**

**Figure S2：RNF216 regulated GN11 cells migration independent of Arc**

A. Depletion of RNF216 upregulated Arc protein level in GN11 cells. GN11 cells were transfected with siNC or siRNF. The protein levels of RNF216 and Arc were detected by immunoblotting. ACTIN was used as a loading control. B. Efficient depletion of endogenous Arc with siRNAs. C. Representative images of GN11 cells from transwell assays. Scala bar=50μm. D. Depletion of Arc did not affect the impaired GN11 cells migration induced by RNAi of RNF216. Data is shown as the mean ± SEM of three independent experiments, ***P<0.001, unpaired *t test.*


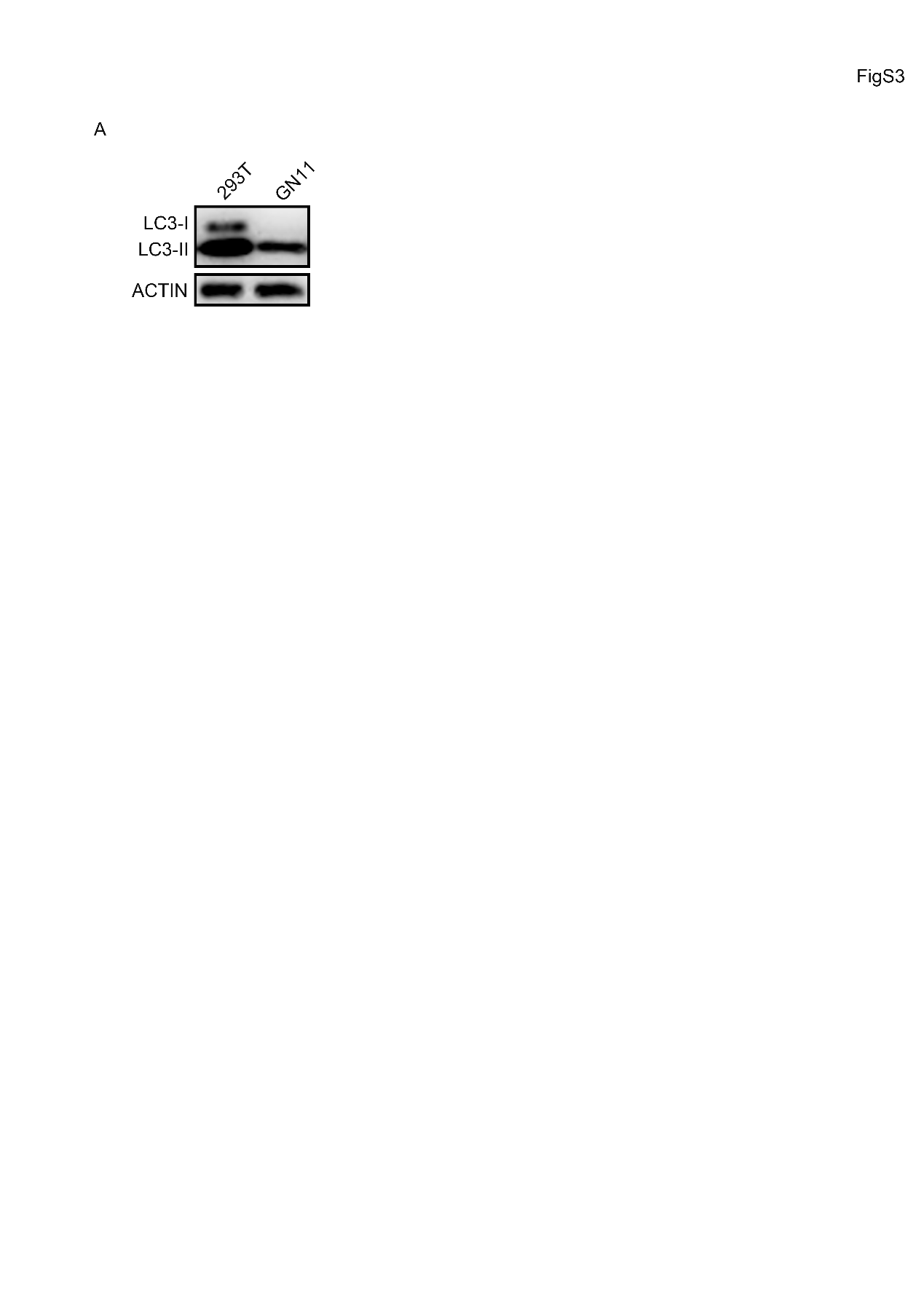


**Figure S3: LC3 antibody can only detect LC3-II in GN11 cells.**

1. LC3 band were detected by immunoblotting. ACTIN was used as a loading control.
